# Direct stimulation of somatosensory cortex results in slower reaction times compared to peripheral touch in humans

**DOI:** 10.1038/s41598-019-38619-2

**Published:** 2019-03-01

**Authors:** David J. Caldwell, Jeneva A. Cronin, Jing Wu, Kurt E. Weaver, Andrew L. Ko, Rajesh P. N. Rao, Jeffrey G. Ojemann

**Affiliations:** 1 Department of Bioengineering, University of Washington, Seattle, USA; 2 Medical Scientist Training Program, University of Washington, Seattle, USA; 3 Department of Radiology, University of Washington, Seattle, USA; 4 Department of Neurological Surgery, University of Washington, Seattle, USA; 5 Department of Computer Science and Engineering, University of Washington, Seattle, USA; 6 National Science Foundation Center for Neurotechnology, Seattle, USA

**Keywords:** Sensorimotor processing, Sensory processing, Somatosensory system, Biomedical engineering

## Abstract

Direct cortical stimulation (DCS) of primary somatosensory cortex (S1) could help restore sensation and provide task-relevant feedback in a neuroprosthesis. However, the psychophysics of S1 DCS is poorly studied, including any comparison to cutaneous haptic stimulation. We compare the response times to DCS of human hand somatosensory cortex through electrocorticographic grids with response times to haptic stimuli delivered to the hand in four subjects. We found that subjects respond significantly slower to S1 DCS than to natural, haptic stimuli for a range of DCS train durations. Median response times for haptic stimulation varied from 198 ms to 313 ms, while median responses to reliably perceived DCS ranged from 254 ms for one subject, all the way to 528 ms for another. We discern no significant impact of learning or habituation through the analysis of blocked trials, and find no significant impact of cortical stimulation train duration on response times. Our results provide a realistic set of expectations for latencies with somatosensory DCS feedback for future neuroprosthetic applications and motivate the study of neural mechanisms underlying human perception of somatosensation via DCS.

## Introduction

Integration of somatosensory feedback into brain-computer interfaces (BCIs) has been shown to improve BCI task performance^1–5^, and is also a consumer design priority for prosthetics users^6^ and potential BCI end users such as individuals with paralysis^7,8^. The study of cortical stimulation for providing somatosensory task feedback has garnered increasing attention because of the realization that the absence of sensory feedback in many current BCIs may limit performance and extensibility^9^. Prior work has shown that humans can respond to direct cortical stimulation (DCS) of the surface of the primary somatosensory (S1) cortex^10–13^, which engenders an artificial sensory percept organized according to standard somatotopy. Recent work has revealed that S1 DCS can be used for somatosensory feedback for closed-loop control in a motor task^14^. Furthermore, DCS has also been shown to induce prosthetic hand ownership^15^. Thus, DCS offers the potential to close the loop in human BCIs by providing a mechanism to encode sensory feedback from an end effector to a user.

While prior work suggests that the integration of somatosensory feedback into a BCI is possible and enhances performance relative to a task without somatosensory feedback, the comparison of human S1 DCS to haptic stimulation has not been well explored. Specifically, given that S1 DCS completely circumvents ascending dorsal column pathways, how human subjects’ response times to DCS differ from response times to natural haptic stimulation has not been examined. This is an important consideration for effective BCI development aiming to integrate cortical stimulation as a method of sensory feedback as response latency invariably constrains feedback loop architecture.

**Figure 1:**
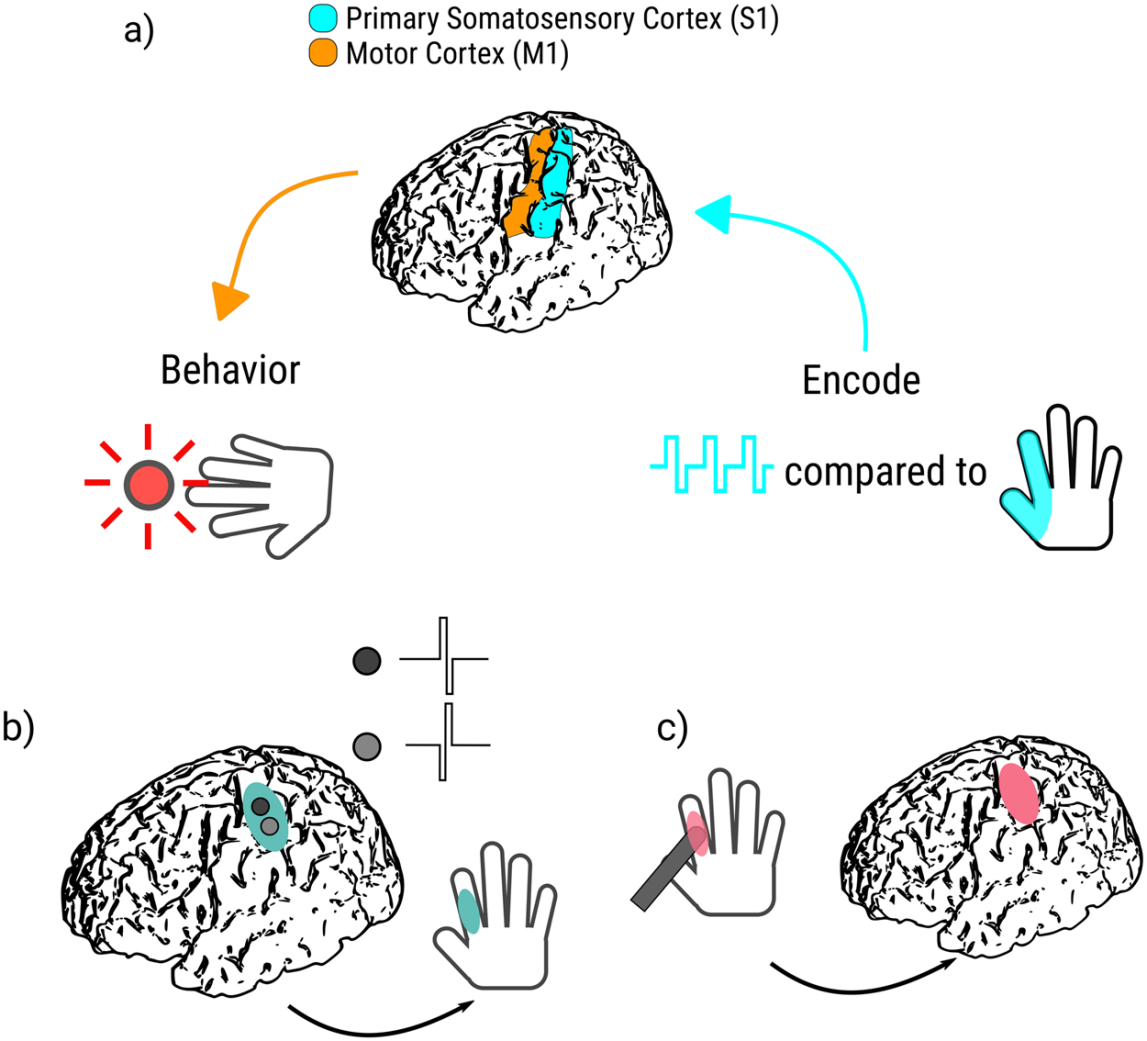
Experimental protocol. (**a**) Here, we test the impact on behavioral performance for native cortical input (haptic touch) compared to artificial feedback (bipolar direct cortical stimulation of primary somatosensory cortex via ECoG electrodes). (**b,c**) Schematic overview of experimental paradigm. (**b**) DCS to S1 hand cortex results in a sensory percept over a specific, consistent location on the hand. (**c**) An experimenter uses a digital touch probe to provide haptic feedback to the same hand location. The subject then responds in both cases as soon as he or she feels sensation in the hand region, using a button held in the opposite hand to perceived sensation.

**Figure 2:**
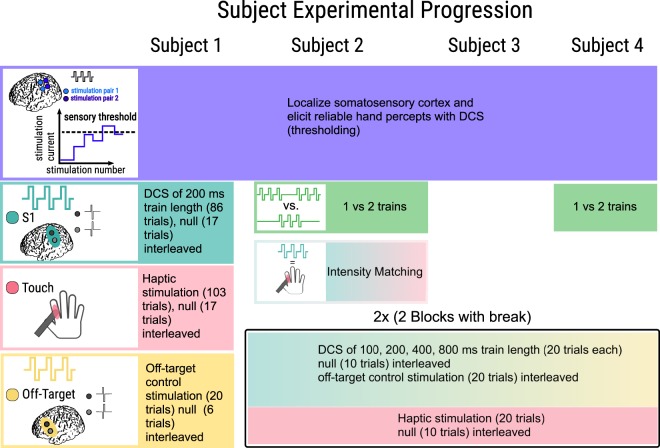
Experimental progression by subject. Each column represents the experimental progression for our four subjects from top to bottom. In all subjects, we localized electrodes which elicited a reliable percept on the hand upon stimulation. We then found a threshold level of stimulation where sensations were elicited, and used stimulation currents above this to ensure reliable perception with 200 ms trains. Subjects 2 and 4 both performed a two-alternative forced choice task of discriminating between one and two trains to confirm our test amplitudes were suprathreshold. Subject 2 then performed an intensity matching experiment in which we identified stimulation levels that elicited approximately the same strength of response as the haptic touch provided by the experimenter. All subjects completed experimental trials after we established the suprathreshold current to use. Subjects 2–4 all had two blocks consisting of 100, 200, 400, and 800 ms trains, interleaved with 20 off-target and 10 null trials, followed by 20 haptic stimuli trials interleaved with 10 null trials.

We asked four subjects to press a button as soon as they perceived either a cutaneous haptic touch to the hand or a percept from S1 DCS via electrocorticographic (ECoG) grids covering the surface of the hand somatosensory cortex (see Fig. 1 for general overview, Fig. 2 for subject specific experimental procedures). We initially hypothesized that direct cortical stimulation, by bypassing the ascending peripheral circuitry, would result in faster reaction times than peripheral haptic stimulation. We additionally hypothesized that subjects would become faster over multiple blocks of DCS as they learned to interpret the signal, and that subjects’ response times to DCS would decrease with longer, sustained train durations relative to shorter trains with a constant stimulation current amplitude.

Remarkably, all four subjects were significantly slower to respond to the S1 DCS than to haptic touch. Additionally, with our two blocks of testing we saw no significant differences between trial types and blocks, suggesting that on a short time scale, appreciable learning was not occurring. In three subjects we tested the train duration hypothesis and found that train lengths as short as 100 ms and up to 800 ms did not significantly affect the response times to the cortical stimulation. We performed off-target testing to serve as a control for the possibility that subjects were responding to stimulation that was applied anywhere in the cortex, rather than directly in somatosensory cortex. This reinforces our testing of electrical stimulation and subsequent activation of primary somatosensory cortex compared to natural ascending peripheral pathways activated through touch, converging on S1. We also included null trials without any stimuli to control for subject suggestibility and response anticipation. Our results shed new light on human perceptual processing of S1 DCS and may direct future studies regarding the application and mechanisms of DCS for both basic neuroscience research and neural engineering applications.

## Results

### Response Times

**Figure 3:**
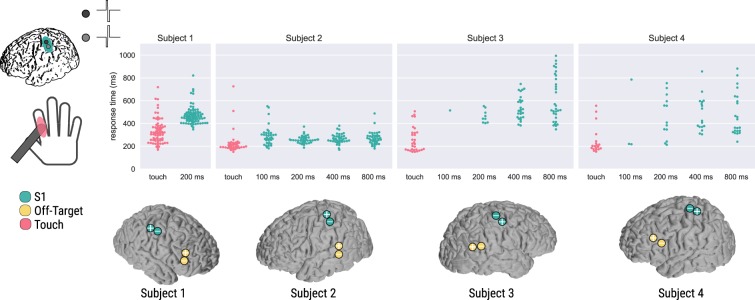
Comparison of reaction times for four subjects and their DCS electrodes. Each dot represents a response time for a given trial, colored by condition. Pink indicates the haptic test condition, while turquoise indicates S1 DCS conditions and electrodes over hand sensory cortex. Subject 1 only received the 200 ms DCS and haptic stimulation conditions, while Subjects 2, 3, and 4 had 100, 200, 400, and 800 ms trains of stimulation applied. The two separate blocks for Subjects 2, 3, and 4 were pooled together for each subject. Off-target DCS control electrodes are indicated in yellow. Electrode locations are based on cortical surface reconstructions for each subject as described in the Methods. Electrodes with a plus symbol (+) indicate anodal-first stimulation, while electrodes with a minus symbol (−) indicate cathodal-first stimulation.

In Subject 1, we compared haptic stimulation to 200 ms trains of S1 DCS with a suprathreshold current amplitude. Haptic feedback elicited a significantly different reaction time as compared to the 200 ms DCS trains (p = 6.105e-16, Fig. 3). The median response time for the S1 DCS trains was 459 ms, while the median response time for the haptic feedback condition was 313 ms (Table 1), consistent with classic tactile reaction times^16,17^. Minimum, 25% and 75% quartile ranges, and maximum response times for all subjects are reported in Table 1. This subject did not perceive off-target DCS, and responded to a single null stimulation trial. In light of the results from Subject 1, we subsequently chose to consider possible effects of S1 DCS train length on reaction times, acquiring and comparing haptic responses to train lengths of 100, 200, 400 and 800 ms with suprathreshold currents in Subjects 2–4.

**Table 1:** Reaction times for each subject and each condition.

Subject	Experimental condition	Minimum (ms)	25% lower quartile (ms)	Median (ms)	75% upper quartile (ms)	Maximum (ms)	Number of trials responded to and within response time bounds
1	200 ms	348	422	459	495	821	81/86
	digital touch probe	169	254	313	374	719	73/103
	null simulation	N/A	N/A	724	N/A	N/A	1/40
	off-target stimulation						0/20
2	100 ms	182	232	277	314	551	36/40
	200 ms	188	235	254	276	372	40/40
	400 ms	169	244	261	288	380	40/40
	800 ms	180	234	265	291	488	40/40
	digital touch probe	151	189	198	228	726	38/40
	null simulation	N/A	N/A	449	N/A	N/A	1/40
	off-target stimulation						0/40
3	100 ms	N/A	N/A	514	N/A	N/A	1/40
	200 ms	403	409	442	494	553	9/40
	400 ms	383	455	515	603	747	26/40
	800 ms	348	466	528	806	994	31/40
	digital touch probe	151	169	222	318	507	30/40
	null simulation						0/40
	off-target stimulation	N/A	N/A	484	N/A	N/A	1/40
4	100 ms	218	219	220	503	786	3/40
	200 ms	213	347	408	595	754	13/40
	400 ms	305	371	423	588	857	17/40
	800 ms	240	334	400	624	882	22/40
	digital touch probe	153	178	201	234	556	19/40
	null simulation						0/40
	off-target stimulation						0/40

In all subjects, cortical stimulation resulted in significantly different reactions times than haptic stimulation (assessed through non-parametric Wilcoxon Rank Sum and Kruskal-Wallis tests). Final column reports the number of trials responded to by each subject across both blocks for each of the trial types given our response time limits of 150–1000 ms, and appropriate signal detection. Response times outside of this range were considered outliers based on expected human performance (see Methods, Data Analysis for details). Blank boxes indicate trial types with no responses.

**Figure 4:**
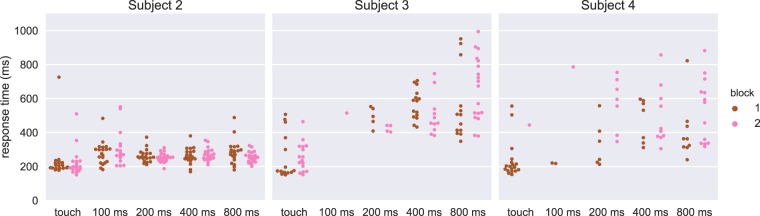
Comparison of the two blocked sessions for three subjects. Each dot represents a response time for a given trial, colored by block. Of note is the non-normality of some of the response timings for different conditions. Additionally, the paucity of responses for Subject 3 to the 100 ms and 200 ms conditions, and for Subject 4 to the 100 ms condition suggests the stimulation level was at or near their perceptual thresholds.

In addition to testing four DCS train lengths for Subjects 2–4, we additionally inserted a rest condition in between two blocks to test for habituation or adaptation (Fig. 4). There were no significant differences between blocks for Subjects 2–4, so we combined them for further statistical analyses. Specifically, for Subject 2, there were no significant blockwise differences between the conditions (p = 0.811, p = 0.715, p = 0.675, and p = 0.0962 for the 100, 200, 400, and 800 ms DCS train conditions, respectively; p = 0.579 for the haptic condition, critical threshold of p = 0.01). For Subject 3, we excluded the 100 ms condition from statistical analyses due to only a single response within one block. Blockwise differences were not significant for any of the other conditions for Subject 3 (p = 0.064, p = 0.087, and p = 0.155 for the 200, 400, and 800 ms DCS train conditions, respectively; p = 0.519 for the haptic condition, critical threshold of p = 0.0125). Similarly, for Subject 4, we excluded the 100 ms condition because of a single response on one block, and two responses on another block. Again, blockwise differences were not significant for any of the other conditions for Subject 4 (p = 0.035, p = 0.669, and p = 0.109 for the 200, 400, and 800 ms DCS train conditions, respectively; p = 0.316 for the haptic condition, critical threshold of p = 0.0125).

For Subject 2, all S1 DCS response times were found to be significantly different than the haptic response times due to statistical differences in medians (p = 3.654e-8 for the 100 ms, p = 7.000e-5 for the 200 ms, p = 2.064e-6 for the 400 ms, and p = 1.866e-6 for the 800 ms DCS train, adjusted p-value threshold = 0.05), while no S1 DCS conditions differed significantly from each other (Fig. 3). The median response times for the 100, 200, 400, and 800 ms DCS trains were 277, 254, 261, and 265 ms, respectively, while the median response time for the haptic feedback condition was 198 ms (Table 1). For this subject we chose off-target stimulation electrodes that had been safely tested during clinical language mapping but used much lower current amplitudes than tested clinically (Fig. 3, Subject 2, off-target electrodes). The subject perceived the off-target stimulation as a vague, non-tactile, and non-localized sensation, and described it as distinct from the DCS sensation. Although he could perceive the off-target DCS, he was able to volitionally choose to not respond to these trial types and did not respond to any of the off-target stimuli within our 150–1000 ms response time window. The subject responded within our time window to a single null stimulus.

For Subject 3 the 200, 400, and 800 ms DCS response times were found to be significantly different than the haptic feedback response times, due to haptic feedback stochastically dominating the reaction times (p = 0.029, p = 5.971e-8, p = 1.290e-10, respectively), while no S1 DCS conditions differed significantly from each other. Subject 3 only responded in one trial with 100 ms S1 DCS trains with a response time of 514 ms, so we excluded statistical comparisons with the other conditions. Median S1 DCS response times were 442, 515, and 528 ms for the 200, 400, and 800 ms DCS trains, respectively, while the median haptic feedback response time was 222 ms (Fig. 3, Table 1). This large difference in medians provides convincing evidence that the cortical stimulation resulted in significantly slower reactions than haptic stimulation. This subject responded within our 150–1000 ms response window once to off-target stimulation, although they did not report being able to perceive the off-target stimulation. The subject did not respond to the null-condition.

For Subject 4 the 200, 400, and 800 ms S1 DCS response times were found to be significantly different than the haptic response times due to a significant difference in medians (p = 1.161e-3, p = 8.803e-5, p = 1.107e-4, respectively), while no S1 DCS conditions differed significantly from each other. Subject 4 responded on only three trials with 100 ms S1 DCS trains with a median reaction time of 220 ms, so we excluded the 100 ms DCS condition from further statistical analysis. Median DCS response times were 408, 423, and 400 ms for the 200, 400, and 800 ms DCS trains, respectively, while the median haptic response time was 201 ms (Fig. 3, Table 1). This subject did not perceive the off-target stimulation or the null stimulation.

For Subjects 1–3, there was no indication of adaptation, nor reported description of the stimulus intensity as weakening and changing throughout the DCS sets. After the first block with Subject 4, however, he verbally described a noticeable decrease in stimulation intensity as the trials proceeded. Therefore on the subsequent block we increased the DCS current amplitude from 1.0 mA to 1.2 mA. The subject again verbally described a decrease in perceived intensity as the trials proceeded during the second block despite the increased current amplitude. This suggests individual differences in adaptation to cortical stimulation, perhaps dependent on parameters such as the electrode location, medication status, subject attentiveness, or amount of cerebrospinal fluid underneath the electrodes.

### Qualitative Assessment

The subjects described the S1 DCS as non-painful, using descriptions such as a “pins and needles” like sensation (Subject 1), a “buzz”, or the feeling of “something brushing” against the skin (Subject 2), “tingling” (Subject 3), and “pulse” or “throb” (Subject 4). These subjective descriptions are in line with previous reports for S1 DCS^10,11,18^. The subjects reliably localized the percept from S1 DCS during the experiment and across blocks (see Table 2 for percept localization). However, the pair of electrodes initially chosen for Subject 2 were not reliably localized, with the subject localizing the percepts from some stimuli to the proximal thumb and some to the proximal palmar area of the fifth finger. Therefore, prior to any experimentation, we selected a different pair of electrodes for Subject 2 that generated a percept which the subject reliably localized to the third finger.

**Table 2:** Subject Demographics.

Subject	Gender	Age	Experiments	Stimulation Current	Coverage and DCS percept localization	Handedness	Seizure etiology
1	Female	21	Cortical Stimulation Digital touch probe Off Target	2500 µA	Right-sided grid; Distal phalange of digit 2	Right	Complex partial epilepsy with multifocal ictal onset and at least 2 distinct epileptogenic areas with seizures arising from right frontal and right temporal regions. No resection/no pathology, VNS implant.
2	Male	37	**Block 1**: Cortical/Off Target interleaved; Digital touch probe **Block 2**: Cortical/Off Target interleaved; Digital touch probe	1500 µA	Left-sided grid; All of digit 3	Right	Focal epilepsy isolated to a left parietal calcified lesion (widespread calcifications eliciting diffuse and severe reactive changes including astrogliosis and microgliosis with unknown origin). Seizures originating from left lateral parietal cortical lesion.
3	Male	26	**Block 1**: Cortical/Off Target interleaved; Digital touch probe **Block 2**: Cortical/Off Target interleaved; Digital touch probe	2000 µA	Right-sided grid; Distal phalanges of digits 3–5	Right	Simple partial seizures from focal cortical dysplasia originating over the right frontoparietal region. No resection–neuropace implant.
4	Male	34	**Block 1**: Cortical/Off Target interleaved; Digital touch probe **Block 1** again: Cortical/Off Target interleaved; Digital touch probe	1000 µA 1200 µA	Left lateral grid; Palmar area near base of digit 1	Right	MRI negative, partial seizures originating from the left mesial temporal area including the anterior temporal pole and hippocampus. Pathology included mild gliosis with leptomeningeal and subpial reactive changes.

This table shows the demographics for all the patients in this study, including experiments completed, stimulation currents used, and the localization of subjects’ percepts, subject handedness, electrode locations, and seizure etiology.

For Subject 2 we attempted to match the perceived intensity of the 200 ms DCS train to that of the haptic stimulation (see Methods, Fig. 2), and although we were able to make their intensities more similar to one another, we were not able to match them completely. As we increased the DCS current amplitude, Subject 2 felt that the percept he experienced both increased in intensity and in the size of the localized area. As a result, during the experiment his perceived intensity of the S1 DCS was slightly less than the perceived intensity of the haptic stimulation in order to keep the localized areas of the sensation similar. Despite matching the sensation intensities as well as possible, Subject 2 described the haptic and cortical stimulation as very distinct from one another. The S1 DCS percept was initially localized to the same region as the haptic stimulation (dorsal side of third finger), but then radiated across the skin.

## Discussion

Our study characterized reaction time differences between cortical and haptic stimulation in four human subjects. Our results demonstrate that response times to cortical stimulation are significantly slower than to haptic stimulation. We additionally demonstrate that cortical stimulation trains of varying lengths do not significantly affect the reaction times for suprathreshold cortical stimulation parameters.

Our results are consistent with a previous observation in non-human primates that intracortical microstimulation of area 1 in primary somatosensory cortex results in significantly slower response times than peripheral stimulation^19^. This delayed response for DCS is counterintuitive at first, as one may suspect that bypassing the ascending peripheral afferents through DCS would reduce the distance traversed by the sensory volley and consequently result in faster reaction times. However, as previously suggested^19^, electrical stimulation may be exciting both inhibitory and excitatory connections in unnatural combinations, driving slower behavioral responses.

In human neocortex, approximately 20% of neurons are interneurons, many of which are inhibitory and contribute to local inhibitory neural circuits^20^. Similarly, in rodent neocortex, approximately 20–30% of neurons are interneurons^21^. This is important when considering the neural response to electrical stimulation, as microstimulation in rodents has been demonstrated to result in a spatiotemporal smear of activity, due to the evoked activity consisting of a combination of fast excitatory responses and inhibitory responses^22^. In addition to an unnatural spatial cortical activation, electrical microstimulation in rodents yields different trends in trial-to-trial variability relative to natural sensory stimuli^23^. Thalamocortical simulations suggest that high levels of synchrony generated by electrical stimuli, which are not seen in natural stimuli, are responsible for this difference in the shape of the trial-to-trial variability curves^23^.

Additionally, electrical microstimulation, as used in the intracortical microstimulation experiments, activates neurons primarily through their axons^24,25^, although other regions of the cell such as the cell body and dendrites may also be activated depending on stimulus polarity and orientation. Non-human primate work using microstimulation combined with fMRI has shown that electrical stimulation may disrupt cortico-cortical signal propagation by silencing output of areas where the afferents are electrically stimulated^26^. This supports the idea that electrical stimulation results in a distinctly different activation pattern, which may explain a less optimal (and longer response time) reaction to electrical stimuli compared to natural haptic stimulation. Other hypotheses for the delayed response to S1 DCS include the possible need for downstream amplification, from a region such as the thalamus, that is initially skipped via S1 stimulation^19^, or the possibility that surface stimulation is unable to directly stimulate deeper primary somatosensory areas, including area 3b where direct intracortical microstimulation has been shown to elicit similar reaction times to haptic stimulation during a discrimination task in non-human primates^27^.

Recent work in computational modeling regarding subdural cortical stimulation in humans suggests that bipolar stimulation at our current levels is unlikely to activate pyramidal neurons directly in the deeper areas of the sulci, and rather, the primary activation of neurons occurs in Brodmann area (BA) 1 on the surface of the cortex^28,29^, and possibly the superficial aspects (towards the crown of the gyrus) of BA3b. Area 3b, where the majority of thalamocortical connections are thought to project^30^, is likely sparsely activated, while Area 3a is in the deepest part of the sulcus^31^, and is activated even less. Therefore, the lack of our ability to effectively target BA3 may partly explain the delayed reaction times to DCS relative to natural haptic touch.

Early cortical stimulation work in elderly dyskinetic patients^12^ suggested a 500 ms stimulation train was required for consistent perception of DCS with a liminal, or near-threshold, current amplitude. Later work in epileptic patients demonstrated that a 250 ms stimulation train could elicit conscious perception with near-threshold current amplitudes^13^. Furthermore, Ray *et al*. illustrated the inverse relationship between DCS train duration and the current amplitude required for perception, with current thresholds increasing as the train durations decreased^13^. We observed a similar phenomenon in Subjects 3 and 4, where for a fixed current, shorter train lengths did not elicit conscious percepts. These two subjects’ inability to reliably respond to the 100 ms train duration condition, suggests that we may have been using a stimulation current amplitude that was too low to reliably discern trains lengths under 200 ms (the train length used for perceptual thresholding) at a fixed amplitude. Additionally, Subject 3 perceived fewer of the 200 ms DCS trials than the 400 ms or 800 ms DCS trials, suggesting that we were stimulating close to the threshold train duration and intensity parameters.

In contrast to Subjects 3 and 4, Subject 2 reliably discerned all of the stimulation trains and had much faster reaction times. In this case we seemed to be operating far above the minimum current threshold necessary for the various DCS train lengths tested. As Subject 2 was the only subject for whom we attempted DCS/haptic stimuli intensity matching (see Methods), we used a current amplitude that was notably greater than the subject’s perceptual threshold (roughly 750 µA greater). The other subjects completed the task with current amplitudes that were only roughly 250–500 µA above their perceptual thresholds. Stronger intensity stimuli are known to produce faster response times^16^, and it is possible that, to a degree, more suprathreshold DCS currents may lead to faster response times, but further experimentation is necessary to examine this hypothesis.

Human tactile, perceptual mean reaction times from one study in untrained, healthy volunteers have been found to vary between 210 and 400 ms^17^, but can range down to 140–150 ms with practice for certain individuals^16^. Reaction times for individuals tend to stay relatively constant between ages 25 and 60^16^. As our patients’ ages (21, 37, 26, 34) are close to within this range, we expect little influence of age on the reaction times. With this as a basis for normal comparisons for our untrained subjects, we similarly find a range of different response times to cortical and haptic stimulation, speaking to individual variability. This suggests that for future BCI implementation, an individual’s innate response time may need to be considered in light of variable latencies. That is, if one subject requires on average 500 ms to respond to cortical stimulation, while another subject requires 300 ms, this requires design considerations on the BCI side to account for time differences in the feedback loop.

Response times are also modulated by non-somatosensory features such as visual feedback, arousal, motivation, and attention^16^. In well-practiced healthy subjects, response times based purely on visual feedback are slower than those based on tactile stimuli for a simple reaction time task (approximately 180 ms on average compared to 140 ms, respectively)^16^. The combination of haptic and visual feedback has been shown to result in faster reaction times relative to visual feedback alone for computer-based tasks in healthy human subjects^32^. We controlled for potential effects of visual feedback by having Subjects 2–4 wear a blindfold, and asking Subject 1 to close her eyes. Subjects’ attention may have also affected their response times, but we did not attempt to quantify their attentiveness. Experimenter observation suggests that Subject 2, who had the fastest response times, was the most engaged in the task and approached it with a competitive, game-like attitude. However, we cannot ascertain that Subject 2′s attentiveness affected his response times, and have presented other possible explanations for his faster responses including use of a higher suprathreshold stimulation amplitude compared to those for the other three subjects. Mere observation suggests that Subject 1 was the groggiest and least engaged in the task, correlating with their slowest haptic reaction times. Future studies may consider including a comparison of response times to S1 DCS and haptic stimuli with visual feedback (i.e., eyes open without a blindfold as would be likely in a future application) to understand how visual feedback may modulate response times. As we increase task complexity and move away from a simple reaction time task as performed here, the benefits from additional feedback beyond only visual feedback may become even more apparent.

An additional factor to be explored in the future is the impact of the polarity of the bipolar stimulation used. Due to experimental time constraints we were unable to comprehensively test the effect of anodic relative to cathodic first stimulation at each electrode, but due to the different cortical activation due to the polarity of stimulation, there could be an effect on reaction times and perception^33,34^.

Each of our blocks lasted on the order of 10 minutes, with 5–10 minutes of rest between the blocks. The lack of a consistent, discernible habituation or learning effect suggests that either the sessions were not long enough or frequent enough to elicit learning or habituation, or that subjects were already reacting close to their fastest possible reaction times. We do not claim that repeated training over multiple sessions and days would not show a decrease in reaction time, but rather we are unable with our acute ECoG epilepsy experiments to address this particular question.

In Subjects 1, 3, and 4, the frontal and temporal electrodes used for the off-target stimulation elicited no sensation and were only responded to once by Subject 3. However, in Subject 2 whose off-target stimulation site was over a language area, the subject perceived a vague, non-localizable, sensation of the stimulation. These off-target electrodes had been safely tested during clinical mapping and avoided possible seizure foci. We used current amplitudes much lower than those tested clinically to further avoid afterdischarges and match the suprathreshold stimulation used in the other S1 DCS conditions. Subject 2 described the off-target DCS as distinct from the S1 stimulation conditions, and had no difficulties in responding only to S1 stimulation. This suggests that humans can receive stimulation in multiple cortical regions and distinguish them within short temporal intervals.

An unknown factor in the work presented here is the extent to which DCS of S1 is also impacting ipsilateral M1, and through connections to contralateral M1, motor output. Our subjects are able to perform motor tasks with the hand being stimulated concurrently, suggesting that there is not grossly visible motor disruption on the ipsilateral or contralateral side. Our subjects also are able to perceive temporally overlapping natural haptic stimulation and DCS at the same spatial location, suggesting that there is not global inhibition or cortical jamming. However, we do acknowledge that some of the delay observed could indeed be due to some potential motor disruption from charge spread. This study does not serve to address this, but rather, presents data revealing significant delays in the timed response to S1 DCS with respect to natural touch. This effect may possibly be due to a delay in conscious perception of the DCS or in the motor output pathway, which has implications for neuroprosthetic and closed loop BCI design.

### Outlook

Our results, while elucidating aspects of human perceptual processing of S1 DCS, demonstrate a need for further exploration of the neural mechanisms underlying the reaction time differences between S1 DCS and haptic stimulation. We found, in four human subjects, that response times to cortical stimulation are significantly different than to haptic stimulation. The fact that there appears to be a significant delay in cortical processing and subsequent response after DCS does not preclude ECoG stimulation from being a promising modality for feedback in a neuroprosthetic application. Rather, this highlights the importance of understanding variables such as human reaction time for neuroprosthetic applications and appropriately designing devices to account for these temporal delays. Our ongoing studies are aimed at understanding and potentially speeding up the temporal response to ECoG stimulation by varying stimulation parameters, regions targeted, and waveform shape.

## Methods

### Subjects

Human subjects (n = 4) were implanted at Harborview Medical Center (Seattle, WA) with electrocorticographic (ECoG) grids (2.3 mm exposed diameter, Ad-tech Medical, Racine, WI, USA) for acute clinical monitoring of intractable epilepsy prior to surgical resection. ECoG grid placement was determined solely based on clinical needs without consideration of research benefits. We conducted all DCS studies after subjects were back on their anti-epileptic medications, after approximately one week of clinical monitoring. Individual patient demographics, including side of electrode implantation and subject handedness, can be found in Table 2, with their corresponding cortical reconstructions and DCS electrode positions shown in Fig. 3. Epileptic foci are also identified in Table 2, to illustrate that we expected neurotypical somatosensory cortical processing for our reaction time task. All patients gave informed consent under a protocol approved by the University of Washington Institutional Review Board. All research and methods were performed in accordance with the relevant guidelines and regulations.

### Cortical Reconstructions

We performed cortical reconstructions based on a preoperative MRI scan and a postoperative CT scan using previously described techniques^35–37^, and identified a pair of adjacent electrodes over the hand sensory cortex based on these reconstructions and clinical mapping information performed by epilepsy technicians for standard clinical care.

### Stimulation Waveform and Hardware

We delivered stimulation through the TDT IZ2H-16 stimulator and LZ48-400 battery pack (Tucker Davis Technologies, Alachua, Florida, USA) with bipolar, charge-balanced, constant current stimulation trains. DCS trains consisted of 200 Hz biphasic pulses with 200 µs per phase, as such DCS trains were previously found to elicit percepts during S1 stimulation^14^.

### Cortical Stimulation

Subjects’ perceptual thresholds for DCS were determined by incrementally increasing the current amplitude of a 200 ms DCS train in steps of 250 µA from a starting amplitude of 500 µA (Subjects 1 and 2), 1000 µA (Subject 3), or 200 µA (Subject 4) until the subject could perceive the stimulation as indicated by verbal report (Fig. 2). In two subjects (Subjects 2 and 3), the first pair of DCS electrodes that we tried did not elicit a consistent perceptual experience, so we tried a different pair of electrodes and again found the perceptual threshold (Fig. 2). Due to experimental time constraints, we only comprehensively tested one pair of stimulation electrodes. During our screening tests we swept through different electrode pairs to choose the pair and stimulation polarity that most reliably produced recognizable percepts localized to the hand. Once we found this pair of electrodes for a given polarity, we conducted all remaining experiments for the day with that bipolar configuration to maximize the number of trials we were able to acquire.

We first determined subjects’ stimulation electrodes and perceptual current thresholds as described above, and then used a suprathreshold current amplitude during the experiment for all DCS conditions (Table 2). To ascertain a suprathreshold stimulation current amplitude, we required two subjects (Subjects 2 and 4) to correctly identify, in ten sequential two-alternative forced choice (2AFC) trials, whether one or two 200 ms DCS trains with a suprathreshold current amplitude were delivered before proceeding from the perceptual thresholding to the response timing experiment (Fig. 2). This demonstrated that the subjects could reliably perceive the 200 ms DCS trains at that current amplitude. For the other subjects (Subjects 1 and 3), we achieved reliable discernment of stimulation with a suprathreshold amplitude (250–500 µA above their perceptual threshold) and proceeded with the response timing experiment without conducting the ten sequential 2AFC trials due to time limitations.

For Subject 2, after successfully completing the ten 2AFC trials, we attempted to match perceived intensity between the haptic feedback condition and the 200 ms DCS train condition by increasing the DCS current amplitude until the subject felt that the two stimuli were of qualitatively equal strength (Fig. 2). We did not attempt intensity matching in Subjects 1 or 4 due to time constraints and patient fatigue. In Subject 3, we did not attempt intensity matching because DCS elicited relatively weak percepts and raising the current amplitude high enough to match its perceived intensity to that of the haptic stimuli would increase the risk of afterdischarges.

### Haptic Stimulation

We applied haptic feedback with digital touch probes (Karolinska Institute) that time stamped the deflection, and touched the cutaneous region where subjects localized the DCS percepts (Figs 1, 2). An audio signal presented to the researcher via headphones but which was inaudible to the subject, cued the experimenter to apply the haptic feedback. We used the digital touch probes previously^15^ in conjunction with cortical stimulation, and at the time of manufacturing they were calculated to have a touch onset with an average delay of 1.04 ± 0.48 ms (mean ± standard deviation). To account for experimenter variability, and possible hardware changes over time, we measured them again and found them to have a touch onset with a delay of mean 5.24 ± 3.26 ms (mean ± standard deviation) and median 6.45 ms relative to an electrical short circuit (Supplemental Information, Figs S1, S2). The small difference in registered touch onset, if added onto the digital touch probe latencies, does not change our significant effects in total (Supplemental Information, Table S1).

### Experimental Protocol

After determining DCS current amplitudes, we completed one (for Subject 1) or two (for Subjects 2–4) blocks of response timing trials, each separated into a DCS set and a haptic stimulation set (Fig. 2). During the DCS set we delivered DCS train lengths of 200 ms for Subject 1, and train lengths of 100, 200, 400 and 800 ms in the subsequent three subjects (Subjects 2–4). Intertrial intervals of both DCS and haptic feedback were jittered (ranging from 2.5 to 3.5 seconds) to minimize anticipatory effects or rhythmic perception by the subjects. We broke up the DCS and haptic stimulation conditions into separate sets to allow subjects to anticipate and focus on one method of stimulation at a time. We reasoned that interleaving haptic and cortical stimulation within one block would result in a greater degree of uncertainty and error due to perceptual differences between modalities, rather than allowing a comparison between conditions where the subject was acclimated to either stimulation type.

All subjects were instructed to respond as quickly as possible by pressing a button held in their hand contralateral to sensation when they perceived the DCS or haptic sensation. The first subject was instructed not to look at the stimulated hand, while the subsequent three subjects (Subjects 2–4) were blindfolded to reduce potential confounds of visual distraction.

### Off-target control stimulation

As a control, we also delivered off-target stimulation to a region outside of S1 during the DCS experimental set. This was to ensure that the responses were specific to DCS of S1, rather than a response to general, non-targeted DCS. For the off-target stimulation electrodes, we chose two electrodes that would be safe for bipolar stimulation based on prior clinical mapping and knowledge of the subjects’ epileptic foci. We used a 200 ms DCS train length and the same suprathreshold current amplitude for off-target stimulation as we used for S1 stimulation. As detailed below and in Fig. 2, Subject 1 completed a third set after the DCS and haptic sets with this off-target control stimulation. For Subjects 2–4, we interleaved off-target stimulation with the on-target, S1 stimulation during the DCS sets.

### Subject 1 trial progression

In Subject 1 during the DCS set, we delivered 86 trials of 200 ms trains of stimuli with 17 trials of null stimuli (i.e., no stimulation as a control) interleaved in a random order. In the haptic set, we delivered 103 trials of haptic touch, again with 17 interleaved null trials. During the third and final set, we delivered 20 trials of off-target stimulation, interleaved with 6 null trials (Fig. 2).

### Subjects 2–4 trial progression

For Subjects 2–4, we first delivered a DCS stimulation set based on stimuli timing and conditions from a pre-generated file that randomly interleaved 20 trials each of 100, 200, 400, and 800 ms train-length S1 DCS trials with 10 null trials and 20 off-target DCS trials, for a total of 80 S1 DCS trials and 30 control trials. Next during the haptic set, we provided 20 trials of haptic stimulation through the digital touch probes, with 10 null control trials randomly interleaved. After a brief rest period (5–10 minutes), we proceeded to a second block of cortical and haptic stimulation sets (Fig. 2).

### Data Analysis

We performed all data post processing and analysis in MATLAB and Python with custom scripts. To calculate the response times in the DCS conditions we took the temporal difference between the onset of the stimulation train and the subject’s button press, while for response times in the haptic feedback condition, we calculated the difference between the registered timing of the deflection of the digital touch probe and the subject’s button press. We identified and excluded outliers as trials with reaction times slower than 1 second and faster than 150 ms from further analysis, as faster responses are unlikely for untrained human subjects^17^, and slower ones more likely represented a decrease in attention to the task rather than a true response time Additionally, we did not consider trials where either the button did not respond appropriately to the subject’s press, or the digital touch probe did not register deflection. Table 1 includes how many trials were analyzed for each subject and condition.

Anderson-Darling tests for normality confirmed that the data was not consistently well described by a normal distribution, therefore we proceeded with non-parametric testing. We corrected for multiple comparisons by dividing an alpha value of 0.05 by the number of conditions tested within each subject. Specifically, both conditions for Subject 1 were not normally distributed (p = 2.725e-4 and 1.888e-8 for haptic and 200 ms DCS conditions, respectively). For Subject 2 the 100 ms DCS, 800 ms DCS, and haptic conditions were not normally distributed (p = 9.631e-5, 0.0096, and 1.399e-16, respectively), while the 200 and 400 ms DCS condition failed to reject the null hypothesis of being normally distributed (p = 0.046, 0.194, respectively). For Subject 3 the 800 ms DCS and the haptic conditions were not normally distributed (p = 0.006 and 3.502e-4, respectively), while the 200 and 400 ms DCS conditions failed to reject the null hypothesis of being normally distributed (p = 0.235 and 0.165, respectively). For Subject 4 the 800 ms DCS and haptic feedback conditions were not normally distributed (p = 0.006 and 1.186e-6, respectively), while the 200 and 400 ms DCS conditions failed to reject the null hypothesis of being normally distributed (p = 0.401 and 0.087, respectively). Due to the presence of non-normally distributed groups, we proceeded with non-parametric testing for all subjects, using the non-parametric Wilcoxon Rank Sum and Kruskal-Wallis tests (with Dunn-Sidák corrections for post-hoc comparisons for mean ranks^38,39^) to assess differences between conditions with an alpha significance level of 0.05. To assess blockwise differences, we used Rank Sum tests with Bonferroni corrections, and a base alpha critical level of 0.05.

Further, we tested for equal variances between groups using the Brown-Forsythe test^40^. For Subjects 2 and 4, testing revealed no significant differences in variances between groups, whereas for Subjects 1 and 3, there were significant differences in variances (critical value of 0.05; not significant- Subject 2: p = 0.094, Subject 4: p = 0.0873; significant- Subject 1: p = 0.0113; Subject 3: p = 5.662e-4). Thus, for Subjects 2 and 4 statistically significant differences between conditions from the Kruskal-Wallis and post-hoc tests were interpreted as differences in medians with haptic stimulation being significantly faster than cortical stimulation, while for Subjects 1 and 3, statistically significant differences were interpreted as differences in stochastic dominance of one sample over another^39^.

### Code Availability

Code required to recreate the above analyses are in the following repository. https://github.com/davidjuliancaldwell/responseTimingPaper.git. MATLAB and Python are required to generate the full set of figures and analyses.

## Supplementary information

Supplementary Information

## Data Availability

Data required to recreate the above analyses are in the following repository. https://github.com/davidjuliancaldwell/responseTimingPaper.git.
