# Publisher Correction: Direct stimulation of somatosensory cortex results in slower reaction times compared to peripheral touch in humans

**DOI:** 10.1038/s41598-019-55968-0

**Published:** 2019-12-27

**Authors:** David J. Caldwell, Jeneva A. Cronin, Jing Wu, Kurt E. Weaver, Andrew L. Ko, Rajesh P. N. Rao, Jeffrey G. Ojemann

**Affiliations:** 1 Department of Bioengineering, University of Washington, Seattle, USA; 2 Medical Scientist Training Program, University of Washington, Seattle, USA; 3 Department of Radiology, University of Washington, Seattle, USA; 4 Department of Neurological Surgery, University of Washington, Seattle, USA; 5 Department of Computer Science and Engineering, University of Washington, Seattle, USA; 6 National Science Foundation Center for Neurotechnology, Seattle, USA

Correction to: *Scientific Reports* 10.1038/s41598-019-38619-2, published online 01 March 2019

The original version of this Article contained extensive errors in the Reference list.

Reference 26 was omitted from the original Article.

Additionally, Reference 32 was incorrectly listed as Reference 26 and References 33–40 were incorrectly listed as References 32–39.

In the Discussion section,

“The combination of haptic and visual feedback has been shown to result in faster reaction times relative to visual feedback alone for computer-based tasks in healthy human subjects^26^.”

now reads:

“The combination of haptic and visual feedback has been shown to result in faster reaction times relative to visual feedback alone for computer-based tasks in healthy human subjects^32^.”

“Due to experimental time constraints we were unable to comprehensively test the effect of anodic relative to cathodic first stimulation at each electrode, but due to the different cortical activation due to the polarity of stimulation, there could be an effect on reaction times and perception^32,33^.”

now reads:

“Due to experimental time constraints we were unable to comprehensively test the effect of anodic relative to cathodic first stimulation at each electrode, but due to the different cortical activation due to the polarity of stimulation, there could be an effect on reaction times and perception^33,34^.”

In the Methods section, under the subsection ‘Cortical Reconstructions’,

“We performed cortical reconstructions based on a preoperative MRI scan and a postoperative CT scan using previously described techniques^34–36^,”

now reads:

“We performed cortical reconstructions based on a preoperative MRI scan and a postoperative CT scan using previously described techniques^35–37^,”

In the Methods section, under the subsection ‘Data Analysis’,

“Due to the presence of non-normally distributed groups, we proceeded with non-parametric testing for all subjects, using the non-parametric Wilcoxon Rank Sum and Kruskal-Wallis tests (with Dunn-Sidák corrections for post-hoc comparisons for mean ranks^37,38^)”

now reads:

“Due to the presence of non-normally distributed groups, we proceeded with non-parametric testing for all subjects, using the non-parametric Wilcoxon Rank Sum and Kruskal-Wallis tests (with Dunn-Sidák corrections for post-hoc comparisons for mean ranks^38,39^)”

“Further, we tested for equal variances between groups using the Brown-Forsythe test^39^.”

now reads:

“Further, we tested for equal variances between groups using the Brown-Forsythe test^40^.”

“Thus, for Subjects 2 and 4 statistically significant differences between conditions from the Kruskal-Wallis and post-hoc tests were interpreted as differences in medians with haptic stimulation being significantly faster than cortical stimulation, while for Subjects 1 and 3, statistically significant differences were interpreted as differences in stochastic dominance of one sample over another^38^.”

now reads:

“Thus, for Subjects 2 and 4 statistically significant differences between conditions from the Kruskal-Wallis and post-hoc tests were interpreted as differences in medians with haptic stimulation being significantly faster than cortical stimulation, while for Subjects 1 and 3, statistically significant differences were interpreted as differences in stochastic dominance of one sample over another^39^.”

These errors have now been corrected in the PDF and HTML versions of the Article.

