# Supplementary material for: Direct stimulation of somatosensory cortex results in slower reaction times compared to peripheral touch in humans
Source: Sci Rep. 2019 Mar 1;9:3292. doi: 10.1038/s41598-019-38619-2 (PMC6397274; doi:10.1038/s41598-019-38619-2)
Supplement: Supplementary file 1 — Supplementary Information [file 41598_2019_38619_MOESM1_ESM.docx]

**Supplementary information**

**Title**

Direct stimulation of somatosensory cortex results in slower reaction times compared to peripheral touch in humans

**Authors and Affiliations**

David J. Caldwell†*^1,2,6^, Jeneva A. Cronin†*^1,6^, Jing Wu^1,6^, Kurt Weaver^3,6^, Andrew L. Ko^4,6^, Rajesh P.N. Rao^5,6^, Jeffrey G. Ojemann^4,6^

†These authors contributed equally

*Corresponding authors

1. Department of Bioengineering, 2. Medical Scientist Training Program, 3. Department of Radiology, 4. Department of Neurological Surgery, 5. Department of Computer Science and Engineering, University of Washington 6. National Science Foundation Center for Neurotechnology

**Digital touch probe latency**

In order to assess reaction times to natural haptic touch via mechanical means, we measured the latency between contact of the digital touch probe to the surface and the digitally registered contact. Previous literature^1^ and corresponding work with our group demonstrated the digital touch probes to have an average onset latency of 1.04 ms ± 0.48 ms standard deviation. To account for experimenter variability and possible hardware sensitivity changes over time, we characterized the latency in onset of the digital touch probes by comparing the registered digital touch probe output relative to an electrical short circuit that triggered on digital touch probe contact with a surface with 294 touches (Fig. S1).


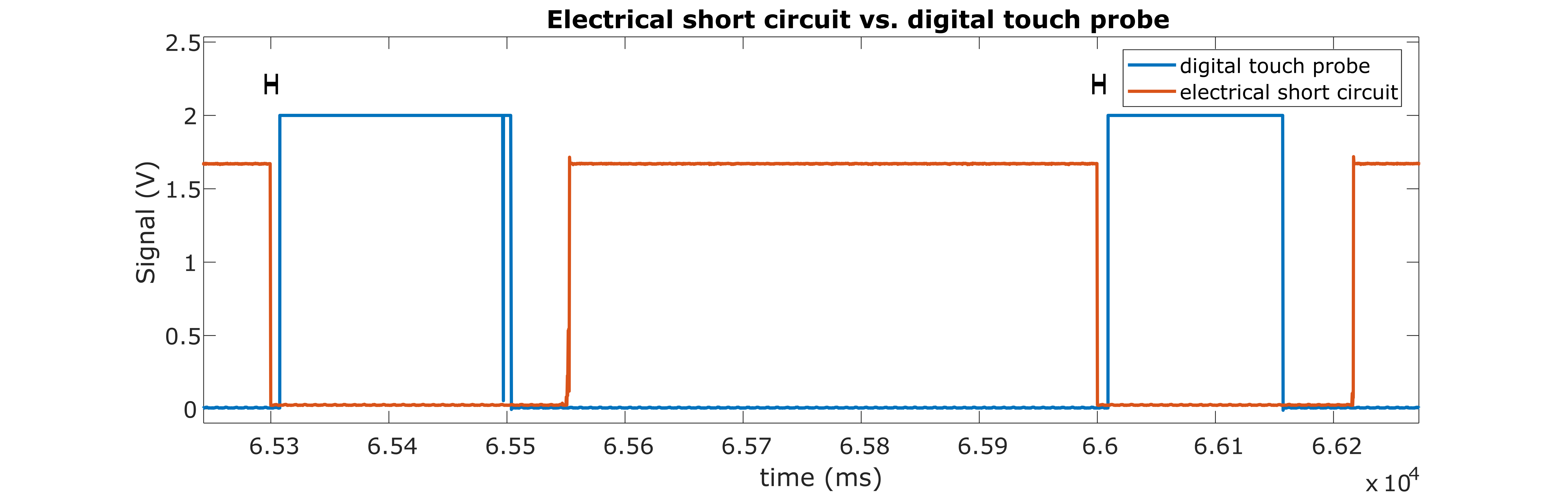


**Figure S1: Example electrical short circuit and digital touch probe signal traces.** Time course of pull-up electrical short circuit relative to the digital touch probe onset. Each touch probe onset was compared to the electrical short circuit that preceded it, as indicated by the horizontal black bars, and these onset latencies were used to generate the distribution of latencies in Fig. S2.

Each trial was registered on the Tucker Davis Technologies RZ5D analog inputs via the front panel, and the two triggered onsets were subtracted from one another to calculate an onset latency. We calculated a mean onset latency of 5.83 ms, a standard deviation of 3.26 ms, and a median onset latency of 5.24 ms (Fig. S2).

**
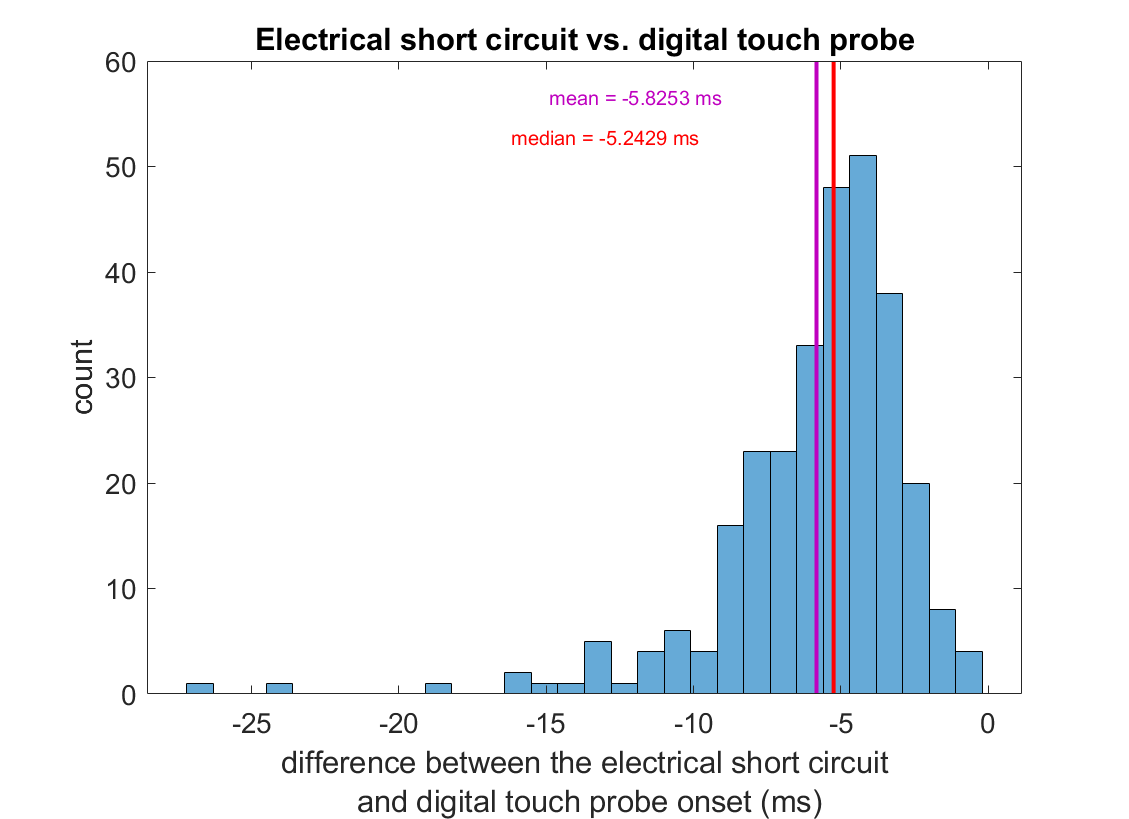
**

**Figure S2: Distribution of onset delays.** Distribution of latencies between an electrical short circuit and the registered application of contact onset using the digital touch probe. Not visualized or included in the calculations are 3 outliers at 63, 68, and 69 ms, due to light or inconsistent experimenter force application.

Based on these measurements, we acknowledge that our response timing results are minimally shifted towards a faster reaction time for natural, haptic touch by an average of approximately 6 ms. Adjusting the current findings for these delays (measured reaction times by the mean onset latency) does not significantly impact our results or interpretations. To specifically illustrate this, we recalculated the statistics after adjustments. These resulting p-values, shown in Table S1, are nearly identical with respect to the originally estimated p-values and thus do not change our conclusions.

| **Subject** | **Experimental** **condition**  **Digital touch probe vs.** | **p-value** |
| --- | --- | --- |
| **1** | 200 ms | 2.101e-15 |
| **2** | 100 ms | 1.834e-7 |
|  | 200 ms | 2.900e-4 |
|  | 400 ms | 9.369e-6 |
|  | 800 ms | 9.046e-6 |
| **3** | 200 ms | 0.033 |
|  | 400 ms | 7.019e-8 |
|  | 800 ms | 1.609e-10 |
| **4** | 200 ms | 2.225e-3 |
|  | 400 ms | 1.125e-4 |
|  | 800 ms | 1.422e-4 |

**Table S1: Adjusted statistics for haptic touch compared to DCS.** Adjusted p-values, by adding on the latency for the digital touch probe to the haptic response times, and subsequently comparing them to the DCS conditions.

**References:**

1. Collins, K. L. *et al.* Ownership of an artificial limb induced by electrical brain stimulation. *Proc. Natl. Acad. Sci. U. S. A.* **114,** 166–171 (2017).
